# Extraction of teeth 11 and 21 due to gemination and space closure with skeletal anchorage in a patient with class III tendency: a case report

**DOI:** 10.1186/s40729-025-00606-w

**Published:** 2025-04-30

**Authors:** Yoana Zinovieva, Moataz Bayadse, Julia Heider, Christina Erbe, Ambili Mundethu

**Affiliations:** 1 Department of Orthodontics, University Medical Center of the Johannes Gutenberg-University, Mainz, Germany; 2 Department of Prosthodontics, University Medical Center of the Johannes Gutenberg-University, Mainz, Germany; 3 Department of Maxillofacial Surgery, University Medical Center of the Johannes Gutenberg-University, Mainz, Germany

**Keywords:** Gemination, Anterior tooth extraction, Gap closure, Skeletal anchorage

## Abstract

Tooth gemination is a dental phenomenon in which a single tooth bud attempts to divide into two, resulting in the formation of a structure that appears as two teeth but originates from the same follicle. This partial separation is often indicated clinically by a groove or depression that suggests the presence of two distinct teeth (Rajeswari M, Ananthalakshmi R. 2011. Gemination-case report and review. Indian Journal of Multidisciplinary Dentistry). The distinction between gemination and fusion plays an important role in treatment planning. If the number of teeth is one less, the tooth is fused and not geminated. In addition, it is assumed in the literature that geminated teeth have a single root canal and fused teeth have two separate root canals (Mahendra et al. in Case Rep Dent. 2014:425343, 2014;Duncan and Helpin in Oral Surg Oral Med Oral Pathol 64:82–87, 1987). The gemination of teeth is relatively rare and occurs mainly in the frontal region of the upper jaw. The prevalence of unilateral tooth gemination in the primary dentition is between 0.01 and 0.04% and in the permanent dentition: 0.05% (Duncan and Helpin in Oral Surg Oral Med Oral Pathol 64:82–87, 1987). Gemination management often requires a multidisciplinary approach and involves several steps ( Rajeswari M, Ananthalakshmi R. 2011. Gemination-case report and review. Indian Journal of Multidisciplinary Dentistry). The orthodontist will then take a thorough medical, dental and family history and perform clinical and radiographic examinations to confirm the diagnosis. Treatment options would include reshaping and restoring teeth with appropriate materials, performing root canal treatment followed by reduction of the mesiodistal width and crown restoration, extraction if the tooth is not suitable for root canal treatment followed by orthodontic space closure or fixed or removable prosthesis if required, transplantation of supernumerary teeth to replace the missing tooth. This case report presents a patient with gemination of teeth 11, 21 and progressive Class III growth tendency. In this case, the malformed anterior teeth were extracted and the gap was closed using skeletal anchorage. Patients with missing central incisors often require a complex interdisciplinary treatment, whether a prosthetic tooth-supported restoration of the missing anterior tooth, single implant, or orthodontic space closure are chosen. Ideally, each alternative should fulfill individual aesthetic concerns, functional requirements, and periodontal tissue health, not only at the end of treatment but also in the long term (Marco in Sem Orthodont 26:1, 2020; Rosa M, Zachrisson BU. Integrating space closure and esthetic dentistry in patients with missing maxillary lateral incisors. J Clin Orthod. 2007; 41(9); Czochrowska ,E.M.,Skaare,A.B.,Stevnik A, Zachrisson, B.U. Outcome of orthodontic space closure with a missing maxillary central incisor;) If gap closure is chosen, it is important to select the correct orthodontic appliance and anchorage especially in Class III patients with sagittal maxillary deficiency.

## Introduction

Dental gemination often manifests clinically and radiographically as fused crowns with or without a longitudinal fissure, a single wide pulp chamber, may include two root canals. The excess of mesiodistal dimensions of the incisors results in poor anterior aesthetics, anterior crowding, insicor rotations, transverse deficiencies, and tooth size discrepancies between the upper and lower jaws, leading to problems in establishing a proper occlusion. Other possible complications include caries and periodontal disease, and in many cases endodontic treatment is complicated due to the abnormal tooth morphology. The solution in such cases is a challenge for any clinician [1, 4, 9].

Even more demanding is the restoration of such teeth in patients with Class III malocclusion and overgrowing mandibles. Class III malocclusion is a complex, three-dimensional skeletal imbalance between the maxilla and mandible, accompanied by varying degrees of dentoalveolar and soft tissue compensation, which may be expressed in a variety of morphological ways. The misalignment may be associated with maxillary growth deficiency (maxillary retrognathia), mandibular growth excess (mandibular prognathism), or a combination of both, together with vertical and transverse malformations [10, 11]. Depending on the skeletal and dental age, orthodontic removable and fixed appliances are available as treatment options. The aim is to enhance maxillary sagittal growth, to inhibit excessive mandibular growth and to achieve physiological overjet and overbite. Especially in cases of maxillary retrognathia, it is crucial to maintain the anteroposterior dimensions of the upper jaw, as any bone resorption in it santerior region can compromise aesthetics, lip support and the stability of overjet and overbite. However, if extraction is required in the anterior region, orthodontic space closure with appropriate biomechanical anchorage could have advantages for the anterior maxillary bone structure and periodontal health [12, 18]. Another positive considertion is that during the mesialization of the lateral incisor, a new alveolar process will form. This new process will have attached gingiva and intact papillae adjacent to the mesialized tooth. These characteristics will be maintained as the dentofacial complex continues to grow [14, 15].

## Case presentation

This case presentation is intended to provide an insight in the treatment of a young patient with skeletal and dental class III who originally had a gemination of the teeth 11 and 21. During the course of treatment, these teeth were removed and the space was closed with a skeletally anchored mesial slider.

## Findings

### Medical history

At the time of first clinical examination at the age of 11.9 years, the patient had a gemination of teeth 11, 21, dentitio tarda, Class III tendency and a horizontal growth pattern.

The Class III tendency was associated with positive OJ and OB and crossbites 12/42, 22/32. The occlusion in the molar region exhibited ¼ premolar width Class III on the right and on the left side, the occlusion in the canine region was Class I on the right and ½ premolar width Class II on the left side with skeletal middle line shift of 2 mm to the left with a forced bite over teeth 22/33.

The general medical history was not pathological. There were no general illnesses, no increased bleeding tendency and no contraindications to performing orthodontic treatment. The profile analysis revealed a straight nasal bridge, enlarged nasolabial angle (129°), incompetent lip closure, reduced mentolabial fold.

### Extraoral findings

The patient showed an enlarged lower third of the face and widened buccal corridors.

**Fig. 1:**
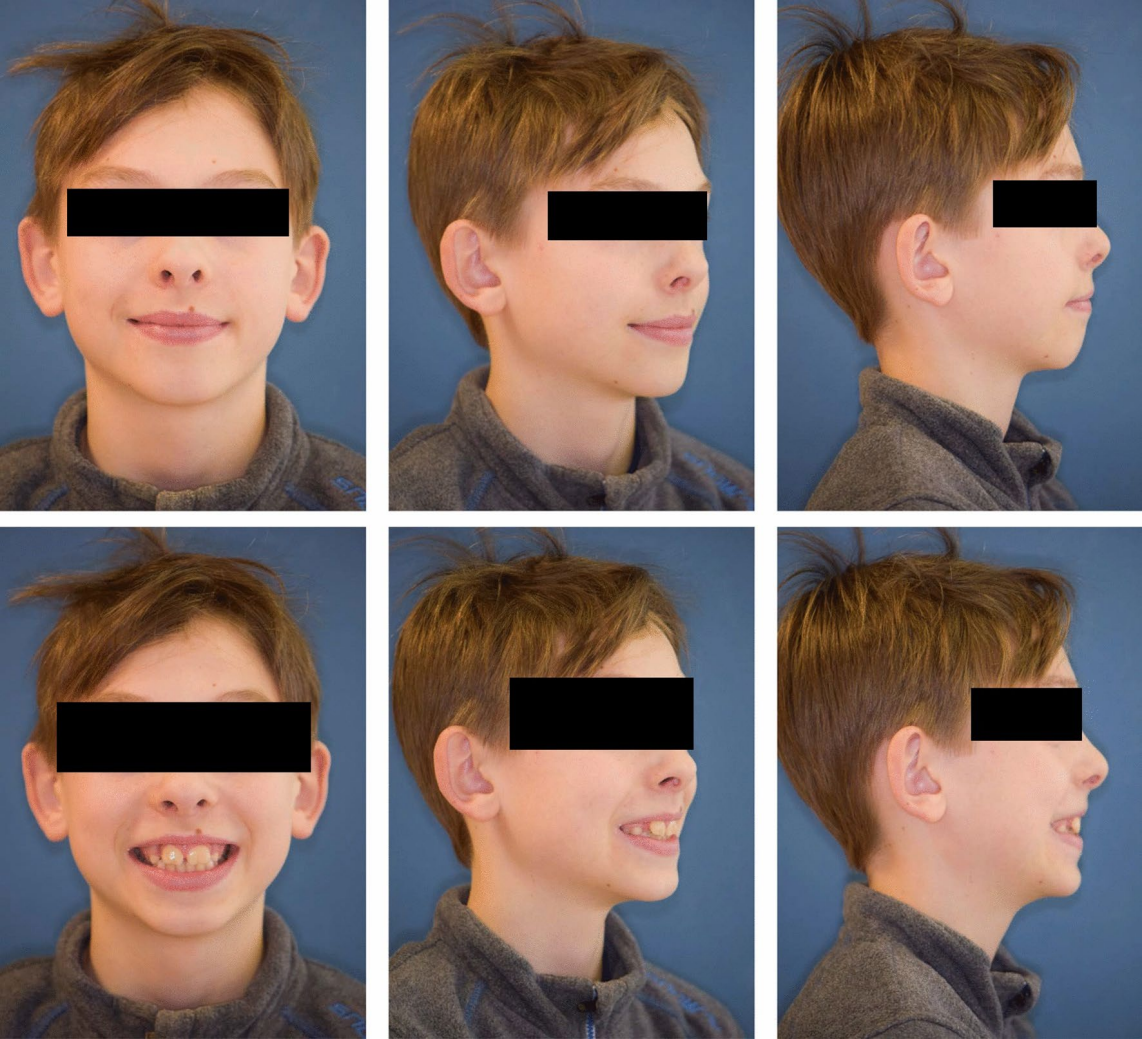
Extraoral findings of the patient before interdisciplinary treatment **a** frontal view, 45° view, right side profile view **b** Final findings extraoral findings: frontal view, 45° view, right side profile view

The profile was slightly convex, with a positive lip staircase, increased nasolabial angle and a straight nasal bridge (Fig. 1).

### Intraoral findings

**Fig. 2:**
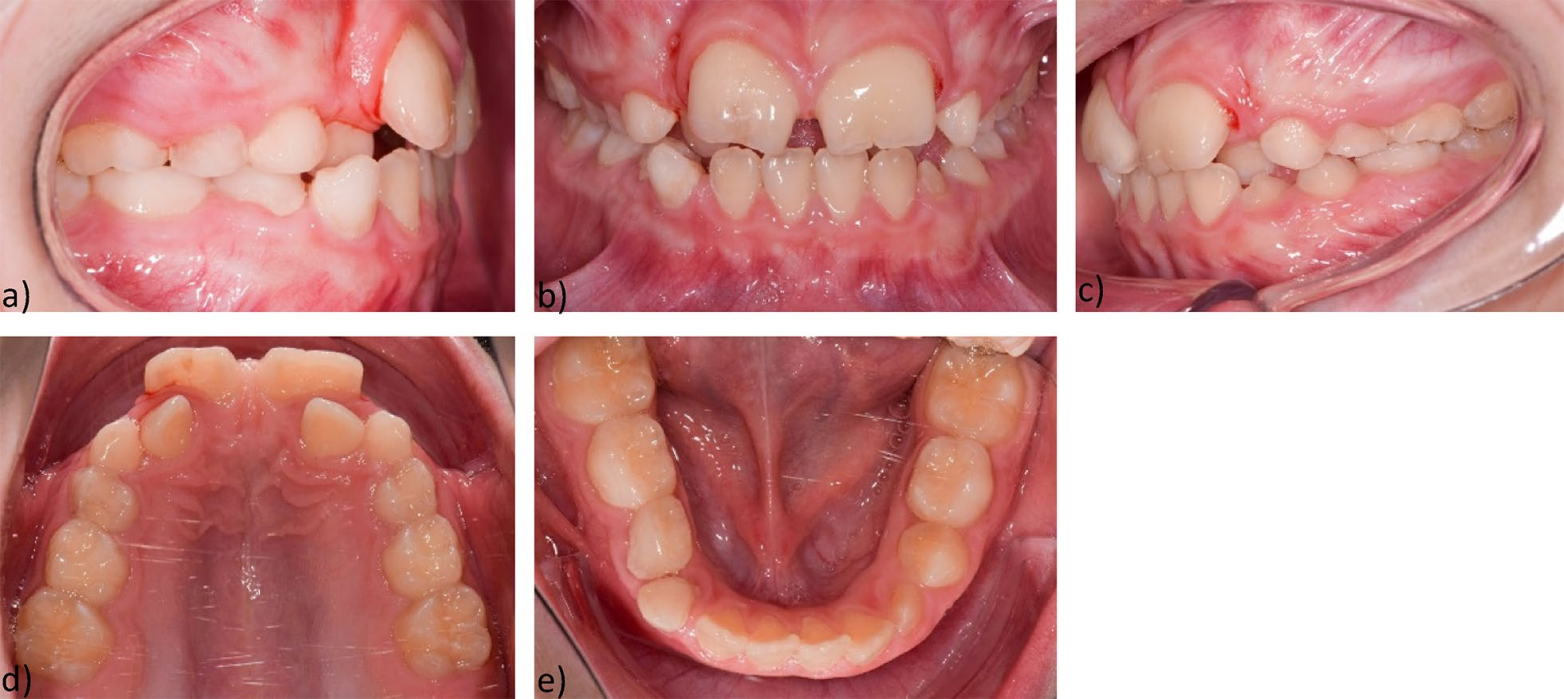
Intraoral findings before treatment **a** Intraoral right-side view **b** Intraoral frontal view **c** Intraoral left-side view **d** Maxillary occlusal view **e** Mandibular occlusal view

Intraorally, a permanent dentition was found with good oral hygiene. Teeth 11, 21 were extracted before the start of the main treatment (Fig. 2).

The maxilla showed a transversal anterior dental arch narrowing, narrow position of the orthoaxially positioned incisors, loss of space and palatal position of 12, 22, diastema divergens 3 mm, various rotational and tilted tooth positions. Arch length discrepancy was: -4 mm. Bolton discrepancy was in range.  

**Fig. 3:**
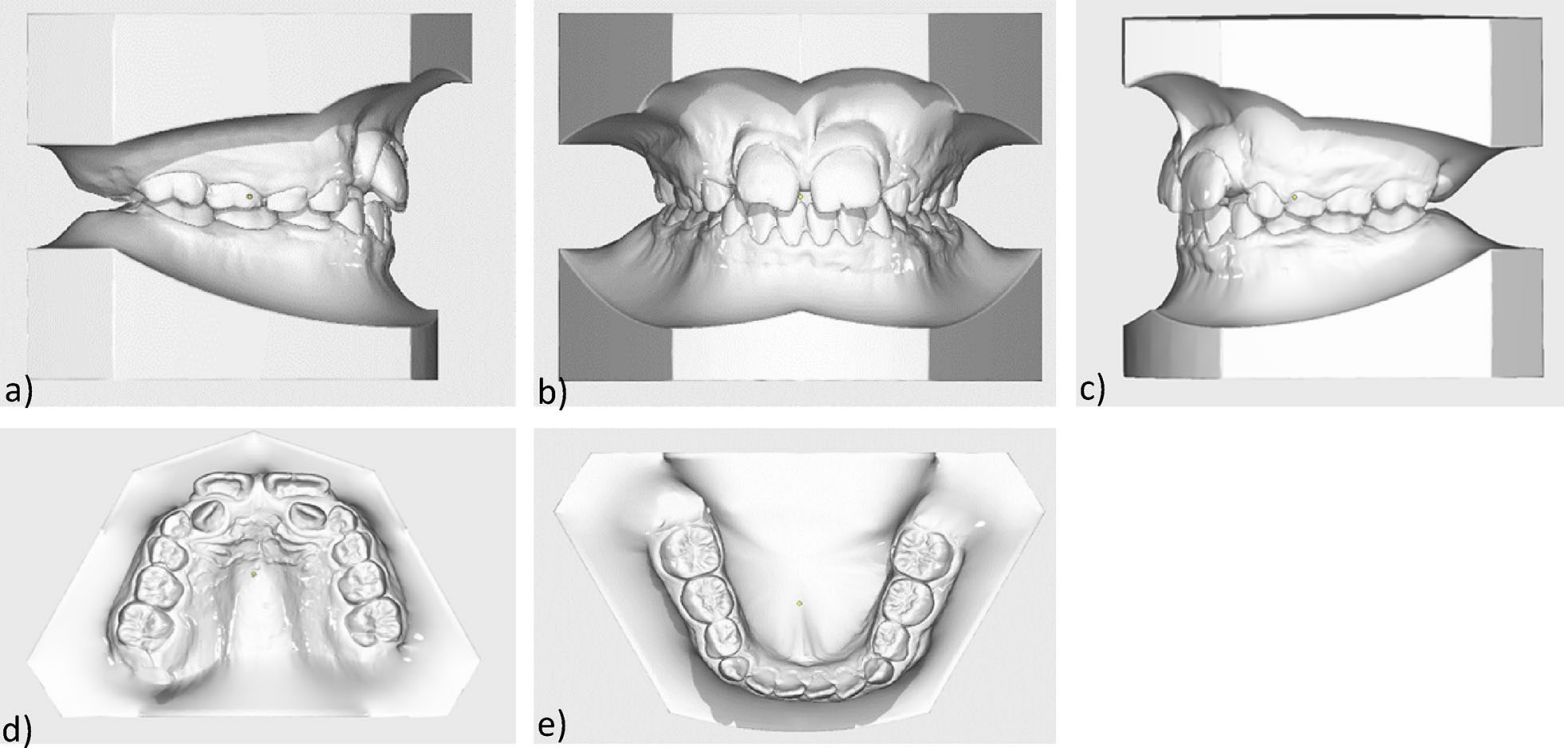
Initial models: **a** Intraoral right side-view **b** Intraoral frontal view **c** Intraoral left-side view **d** Maxillary occlusal view **e** Mandibular occlusal view

The lower jaw also showed a transversal anterior dental arch narrowing, crowding of the orthoaxially positioned anterior teeth, various rotational and tilting tooth positions, mesiorotation 11, distorotation 31, 32, pronounced Spee- and Wilson- curves, arch length discrepancy was -1 mm (Fig. 3).

### Radiological findings

**Fig. 4:**
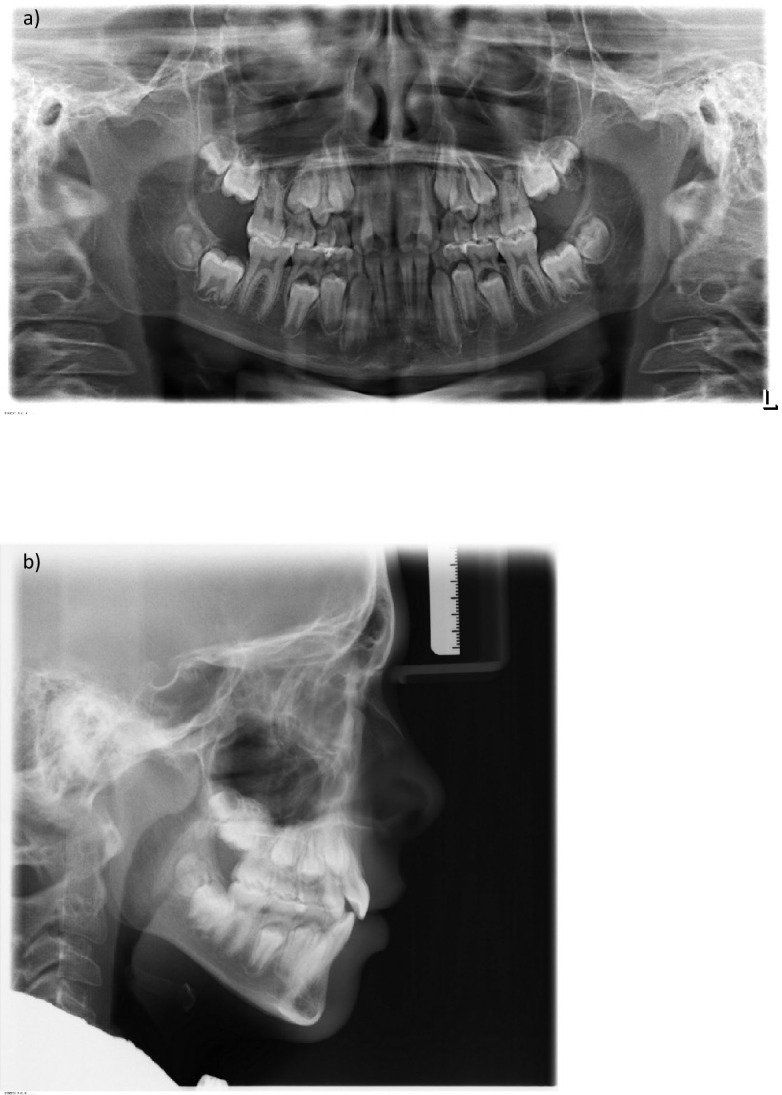
**a** Panoramic X-ray at the start of treatment **b** lateral cephalogram at the start of treatment

The panoramic radiograph showed a late mixed dentition. All permanent teeth, including sapientes, were in place, macrodontia 11, 21, narrow germinal position of the maxillary premolars with a narrow central apical base, asymmetry of the condyles on the right and left sides. The maxillary sinuses were pneumatised. No radiological abnormalities were detected (Fig. 4a).

The cephalometric analysis revealed a neutral jaw base relationship with mesial tendency in the anteroposterior plane.

The mandible presented a normodivergent inclination which, together with the normodivergent inclination of the maxilla, resulted in a neutral jaw relationship with a horizontal growth tendency.

The upper and lower incisors were orthoaxial, with maxillary incisor angle to the nasal line (I/NL) at 102.6° and mandibular incisor angle to the mandibular line (I/ML) at 95°, respectively. Accordingly, the interincisal angle was within the normal range, measuring 133.3° (Fig. 4b).

A dental crossbite was present anteriorly due to the palatal position of the lateral incisors in the upper jaw.

### Treatment goal

The main aim of the treatment was to extract the incisors in the upper jaw and to close the space anteriorly by mesializing the posterior teeth while maintaining a physiological overjet and overbite. A space closure was preferred to a prosthetic implant due to the young age of the patient.

### Treatment plan

**Fig. 5:**
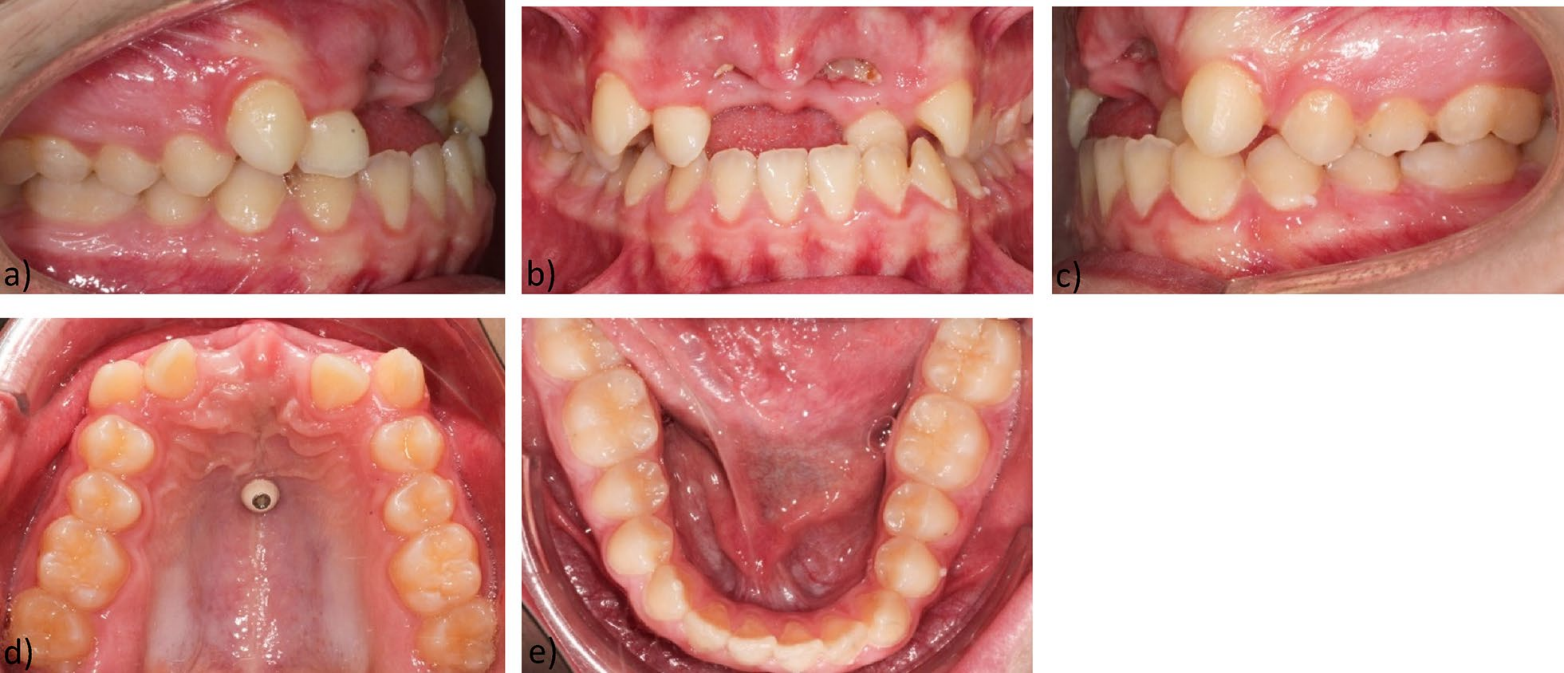
Findings after extraction of gemination 11, 21 **a** Intraoral right-side view right **b** Intraoral frontal view **c** Intraoral left **d** Maxillary occlusal view **e** Mandibular occlusal view

The main treatment plan included extraction of teeth 11, 21 resolution of the frontal crowding, mesialization of the right and left posterior teeth with the assistance of skeletal anchorage along with reshaping of the maxillary dental arch (Fig. 5).

In the lower jaw, the resolution of the anterior crowding was initially planned only with proximal enamel reduction through the entire mandibular dental arch. Due to the increase in prognathic mandibular growth and the lack of space in the anterior region, extraction of a mandibular incisor was performed. A lingual arch was placed on the posterior teeth to allow space closure mainly from anterior while resolving the anterior crowding.

The objectives for the dental occlusion were to set a physiological overjet and overbite, maintain the Class I occlusion in the molar region and set a Class I occlusion in the canine region on the right and maintain a slight quarter-premolar-width Class II occlusion in the left canine region.

### Treatment process

The treatment began with an initial phase in which a removable appliance (plate with transversal expansion screw) was constructed to correct the transversal deficiencies, to reduce the medial diastema and resolve the frontal crowding in the maxilla.

The patient was instructed to optimize his oral hygiene. In addition, speech therapy was prescribed to improve the patient's articulation and restore orofacial balance.

After re-evaluation of the diagnostic records, the decision was made to remove the macrodontic teeth 11, 21 and to close the anterior gap by mesializing the posterior maxillary teeth with a skeletally anchored mesialization appliance.

**Fig. 6:**
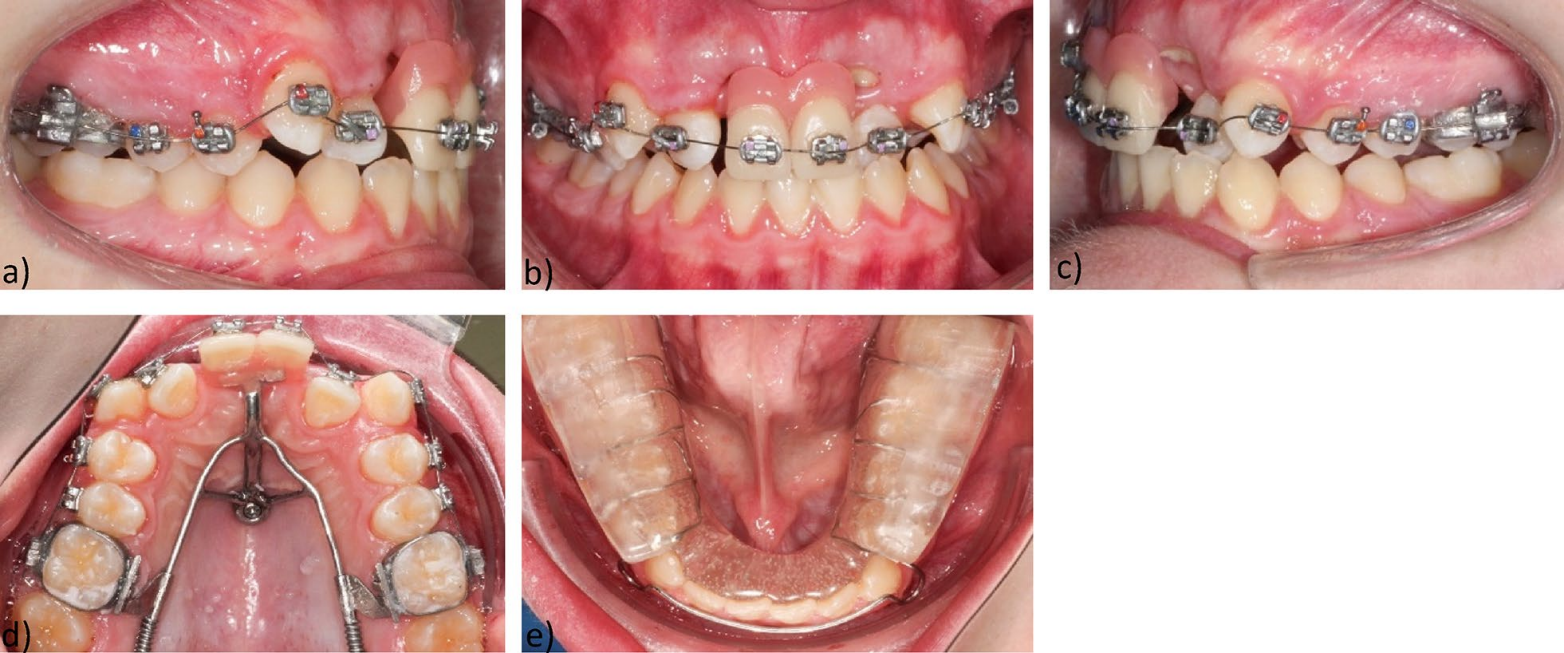
Superstructure for the mesialization of the upper posterior teeth **a** Mesial slider, **b** Prosthetic replacement of the extracted teeth 11, 21 **c** Intraoral left-side view left **d** Maxillary occlusal view **e** Mandibular occlusal view Bite plate to decouple the occlusion

Initially, a palatal implant (Orthosystem, Straumann, Basel, Switzerland) was inserted at the level of the second pair of palatal folds. A multibracket appliance was then placed with bands on 16, 26 and brackets 12–15, 22–25. In order to close the gap of the missing anterior teeth, a superstructure including a mesial slider was placed and activated to mesialize the posterior teeth. In the initial phase of space closure, the missing anterior teeth were replaced with prosthetic teeth for aesthetic reasons. The latter were gradually reduced and adapted to the mesial movement. The mandibular appliance was reduced to a bite plate to decouple the occlusion and allow tooth movement (Fig. 6).

During the follow ups a successful mesial movement of the posterior teeth in the upper jaw was detected. In addition, there was an increase in the prognathic growth tendency and the crowding in the anterior teeth of the lower jaw. One year after insertion of the fixed appliance in the upper jaw a complete diagnostics with subsequent re-evaluation was carried out. Due to the progression of the prognathic growth direction (Wits appraisal: − 11 mm) and the increase in the crowding of the mandible anteriors, the treatment plan was reevaluated.

First a multibracket appliance was inserted in the lower jaw. To resolve the crowding in the mandibular anteriors, an extraction procedure with removal of tooth 41 was performed. As a further therapy in the upper jaw the mesialization of teeth 12 and 22 in the place of 11, 21 and the distal alignement of the canine teeth was planned. It was decided to use teeth 11, 21, 13, 23 as bridge abutments and to replace 12, 22 with pontics. The superstructure was removed in the upper jaw and the abutments were distributed using push to pull mechanism by means of elastics or coil springs. Compression springs were inserted mesially to 13 and 23, and an active tieback from each canine to the molar on the corresponding side were used for successful retraction and distal uprighting of the two canines.

A lingual arch was used to stabilize the posterior segment and prevent loss of anchorage in the mandible during the remaining space closure anteriorly. The patient was instructed to wear Class III and Up and Down elastics for fine adjustment of the occlusion. The multibracket appliance was removed at the same time in both the maxilla and the mandible.

**Fig. 7:**
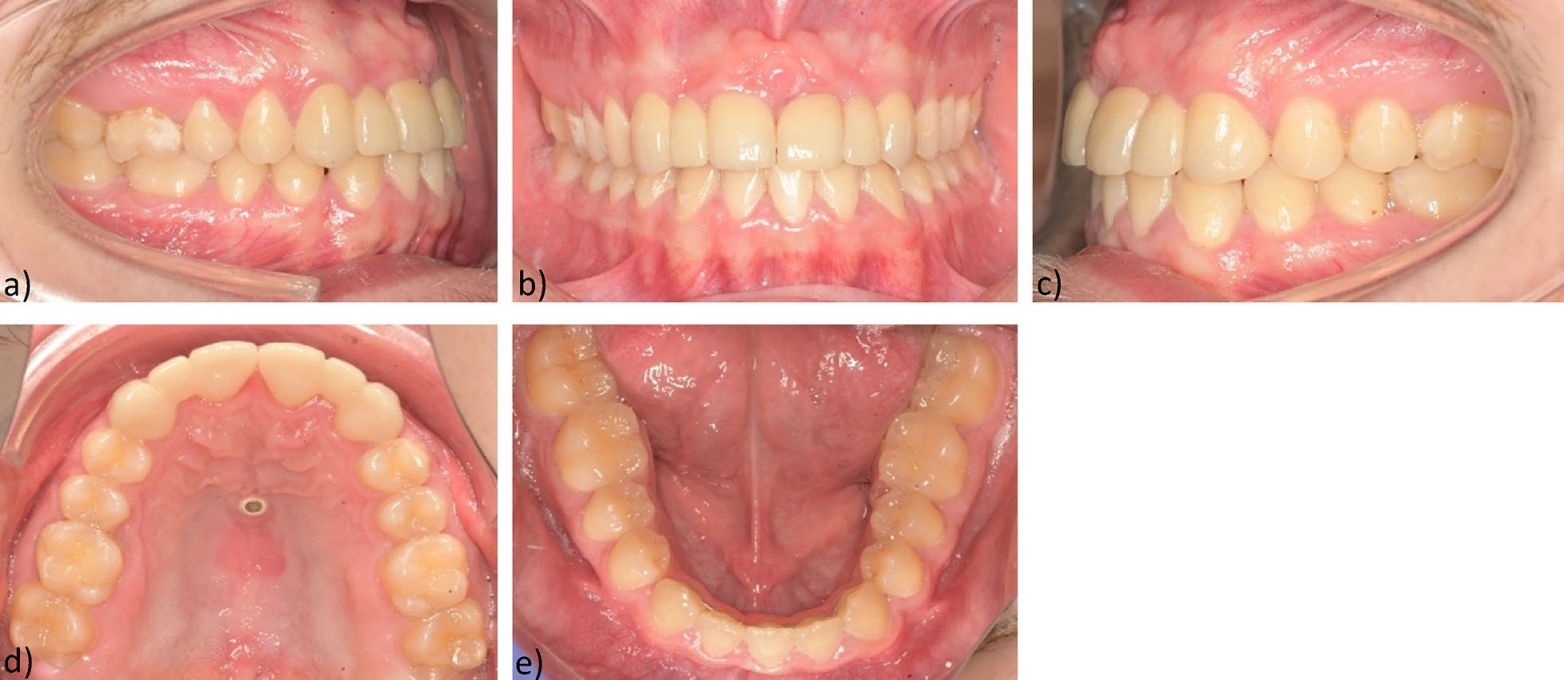
Final result **a** Intraoral right-side view **b** Intraoral frontal view **c** Intraoral left **d** Maxillary occlusal view of temporary bridge 13–23 **e** Mandibular occlusal view of lower retainer 33–43

After removal of the multibracket appliance, a fixed bonded retainer was inserted in the lower jaw. As additional retention, the patient was given a removable retention plate and was instructed to wear it only at night. For the temporary restoration in the upper jaw, a removable dental aligner with replacement teeth 12, 22 and a build-up of teeth 11, 21 was fabricated and adapted (Fig. 7).

**Fig. 8:**
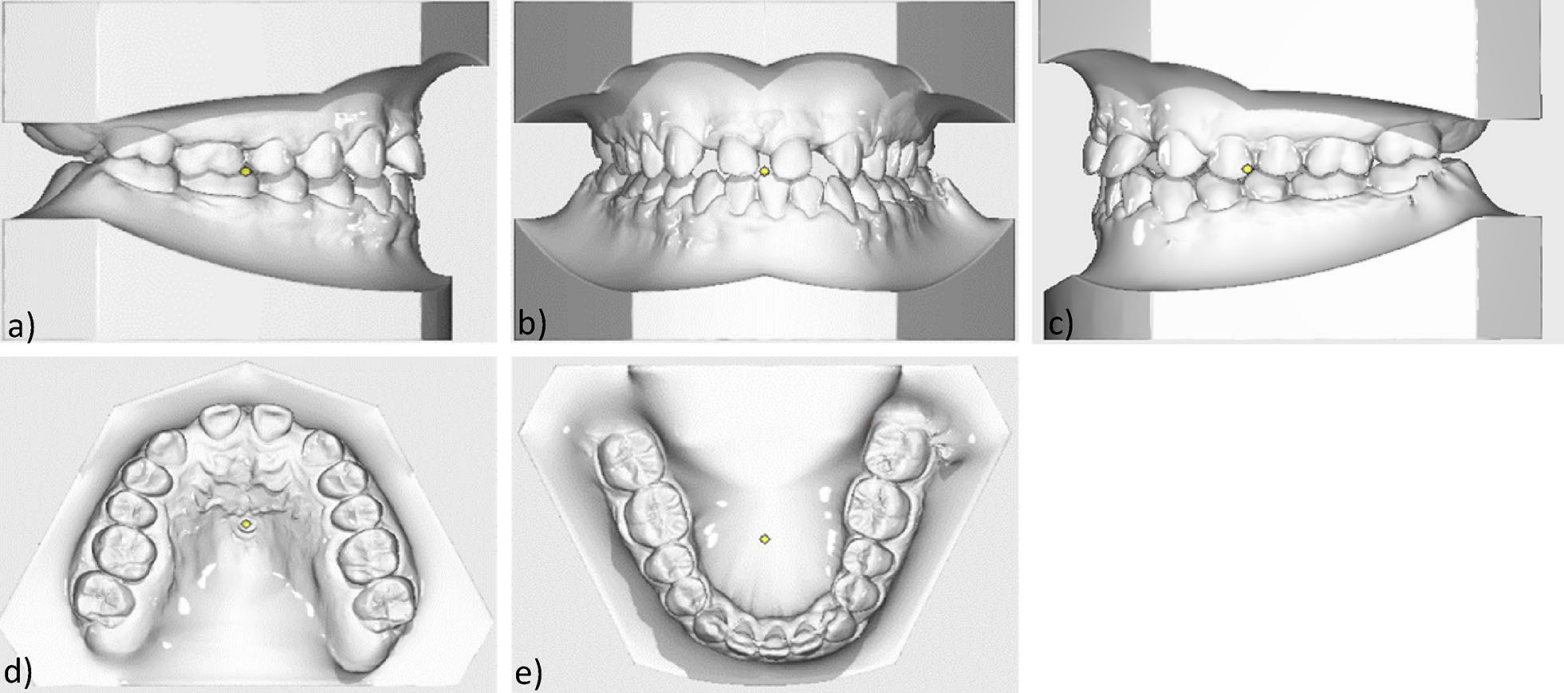
Final models: **a** Intraoral right-side view **b** Intraoral frontal view **c** Intraoral left-side view **d** Maxillary occlusal view **e** Mandibular occlusal view

**Fig. 9:**
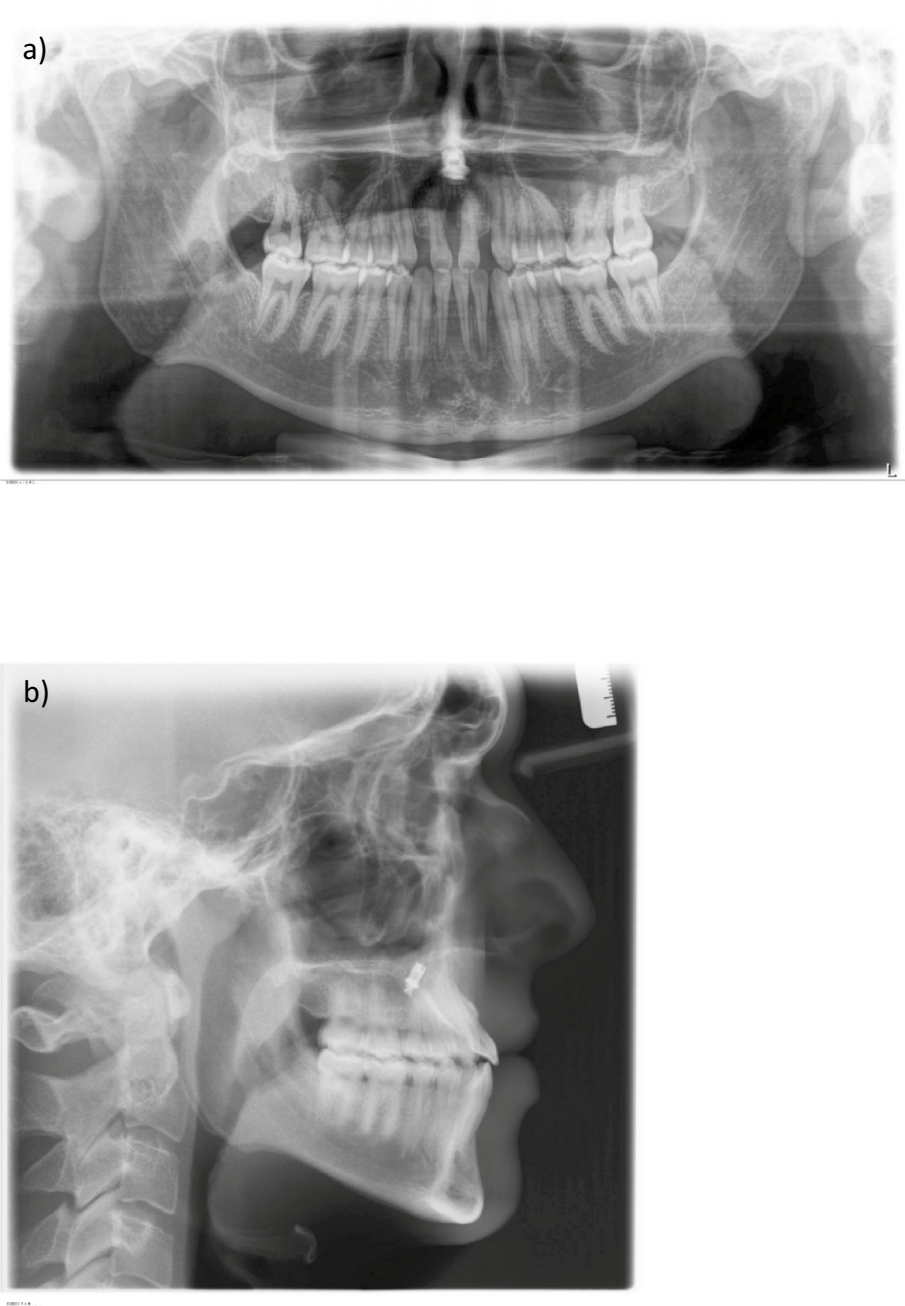
**a** Panoramic X-ray at the end of treatment **b** Lateral cephalometric image at the end of treatment

**Fig. 10:**
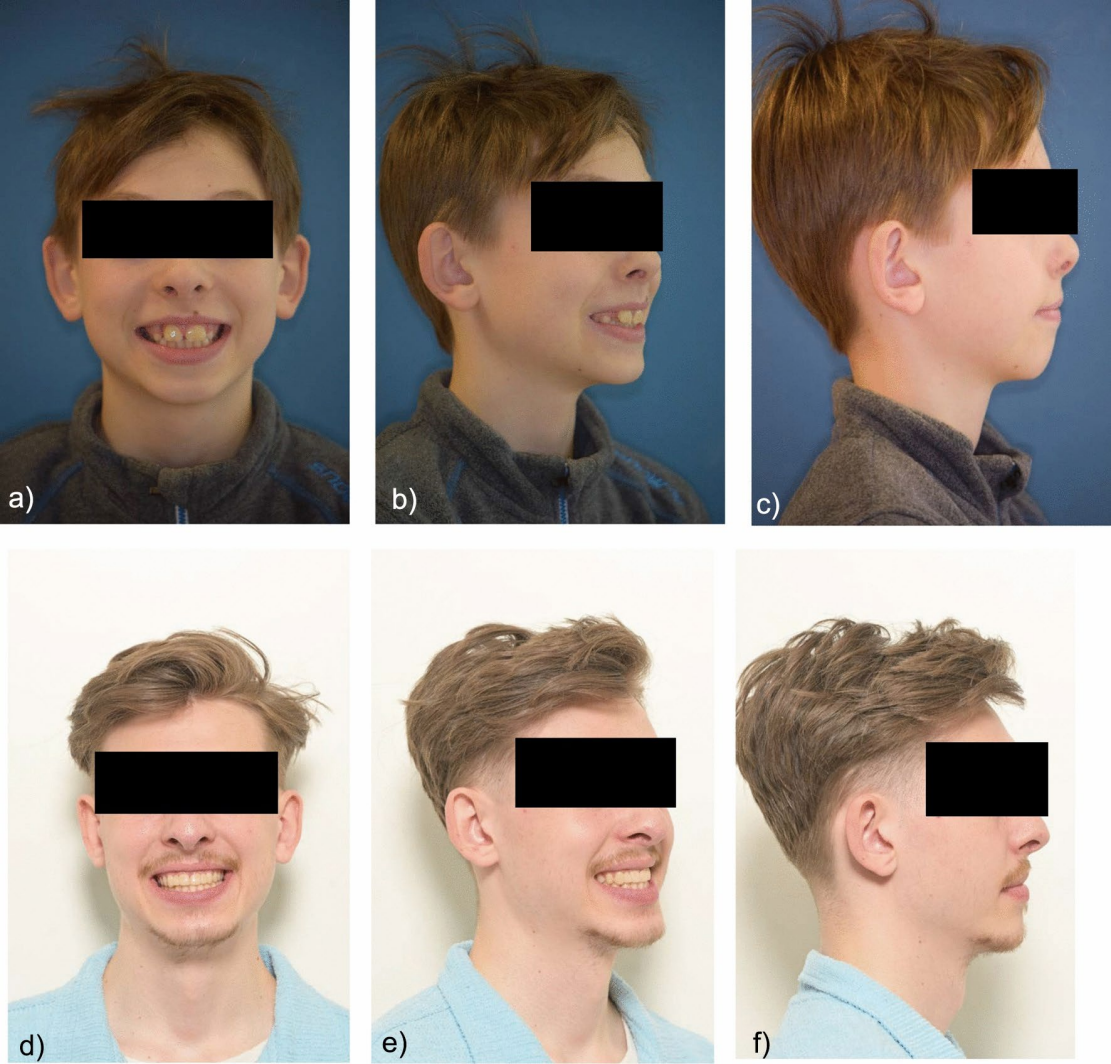
Extraoral findings after interdisciplinary treatment: **a** frontal view, 45° view, right side profile view **b** Final findings extraoral findings: frontal view, 45° view, right side profile view

**Fig. 11:**
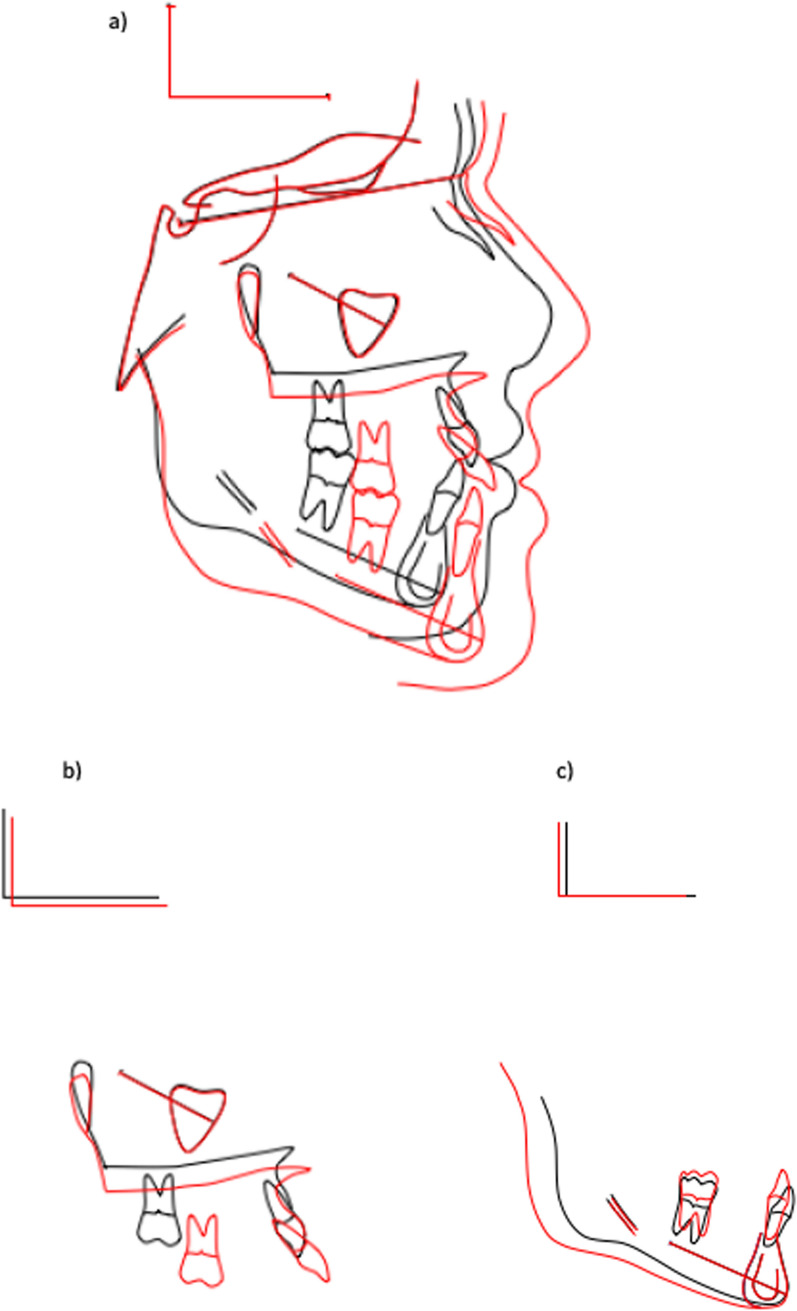
Björk's method of cephalometric superimposition. Black: Pretreatment Red: 2 years post-treatment **a** General superimposition **b** Maxillary superimposition **c** Mandibular superimposition

After completion of the orthodontic treatment, a Class I occlusion was achieved in the molar region on both sides with a physiological overjet and overbite. The anterior cross bite was eliminated and freedom of mandibular movement was attained (Figs. 8,9,10,11).

## Discussion

The main orthodontic issues in this patient were the presence of macrodontic teeth 11, 21 and the progressive mandibular prognathic direction of growth.

At the beginning of treatment, the patient was still growing. For this reason, a removable plate with expansion screw was placed in the upper jaw to correct the transversal deficiency and to allow the normal development of the maxillary dentition.

After careful reevaluation and reconsideration of the treatment plan, a decision was made to extract the abnormal teeth for both aesthetic and functional reasons. One of the major challenges in extraction therapy is achieving healthy periodontal tissues after bone loss in the midline region to meet aesthetic and functional requirements.

A space closure by mesialising the posterior teeth was planned. Orthodontic space closure leads to natural remodeling of new bone formation as the tooth moves through the alveolar bone and creates conditions of improved gingival health [2]. Due to the large number of teeth that had to be moved to close the gap of the missing central incisors and to avoid dentoalveolar side effects such as loss of anchorage, skeletal fixation with a palatal implant was chosen [3].

Another treatment alternative would have been the insertion of implants at the site of the removed teeth 11, 21, which was not carried out due to the young age of the patient and the expected growth.

An endosseous orthodontic implant (Orthosystem, Straumann, Basel, Switzerland) was placed. It consists of a pure titanium implant with a surface-treated screw-shaped endosseous section of 3.3 mm diameter and 4 and 6 mm length. Due to the mechanical properties of the surface, the palatal implant osseointegrates to provide a biomechanically optimal, compliance-independent anchorage for mesialisation of the posterior teeth and space closure anteriorly [16].

To prevent unwanted protrusion of the lower incisors due to the aligning of the dental arch with multibracket appliance associated loss of the positive overjet, treatment in the mandible was only started at a later date.

The increasing lack of space in the lower jaw and the progressive prognathic growth tendency were successfully managed through a camouflage treatment, including the extraction of a mandibular incisor.

Extraction of two premolars was also considered as an alternative solution to adress the anterior crowding. However due to the moderate expression of the crowding and the progressive compensatory retrusion of the anterior teeth, this option was not selected as the preferred treatment. Other undesirable side effects of this alternative in the presence of a neutral growth pattern would be the risk of undesirable bite deepening and the negative impact on the profile.

Another option for treatment of the skeletal discrepancy could have involved a rapid palatal expansion device combined with miniplates or miniscrews in the lower jaw, supported by Class III elastics [17]. However, this approach would have been significantly more invasive for the patient. In cases of poor soft tissue response and unfavorable growth progression, a combined orthodontic approach with maxillofacial orthognathic surgery would have been the only reliable method to establish stable occlusion after growth is complete.

Considering the initial situation, the treatment result was good. Teeth 12, 22 could be harmoniously mesialized into the dental arch. Teeth 13, 23 could be distalized and thus have a good functional occlusion. The maxilla could be successfully re-developed in the transverse dimension. However a slight sagittal deficiency remained in the upper jaw due to the mandibular prognathism tendency and bone loss in the anterior alveolar ridge following the extraction of the macrodont teeth 11 and 21. It was decided to use teeth 11, 21, 13, 23 as bridge abutments and to replace 12, 22 with pontics. Maryland bridges were not chosen as the preferred treatment due to the narrow roots of the lateral incisors and the risk of overloading when used to support the crown of the central incisor [14, 15].

A clear decrease in the SNA angle between the initial and intermediate findings was observed in the superimposition of the cephalometric analysis according to Björk. Most likely as a result of the extraction of the geminated teeth 11, 21 and the associated bone resorption.

A physiologic overjet and overbite and a bilateral Class I occlusion in the molar region were established.

A removable retention device with replacement of teeth 12 and 22 was placed in the maxilla as a temporary solution for aesthetic reasons.

Further vertical settling was accomplished with up-and-down elastics 23/ 33, 34. The subsequent restoration of teeth 11, 21 and the prosthetic rehabilitation of the extraction gaps 12, 22 was performed at the Department of Prosthodontics, University of Mainz.

As a definitive solution, the fabrication of two bridge constructions in the upper jaw was planned and completed.

The patient is very satisfied with the functional and aesthetic outcome.

## Conclusion

The treatment of patients with geminated incisors presents a unique challenge for every orthodontist. When combined with a skeletal Class III malocclusion, a specialized interdisciplinary approach is required for effective patient management. Extraction and gap closure/gap distribution, especially in patients with sagittal deficiency of the maxillary arch, is an excellent aesthetic and functional alternative for bone preservation of the anterior alveolar ridge in the upper jaw in the long term and establishes a sufficient occlusion while maintaining positive overjet and overbite. At the end of the treatment the upper lateral incisors were slightly more protruded to compensate for the skeletal Class III malocclusion.

As mentioned above, a major advantage of space closure is that the treatment result is permanent, although long-term maintenance is required. In this particular case, this is important because the patient with the missing maxillary central incisor is an adolescent. Another advantage of space closure is that it creates a normal gingival topography around the mesially displaced lateral incisors. Intact marginal and interdental gingival contours are difficult to create and maintain with single-tooth implants and ceramic bridges. A third advantage of space closure is cost, as the restoration of the residual space of the lateral incisors could be performed with Maryland bridges and would not require implants [13, 15].

## Data Availability

No datasets were generated or analysed during the current study.

## References

[1] B. W.Neville, D. D. Damm,C. M. Allen,andJ.E.Bouquot, Oral and Maxillofacial Pathology, Saunders, Philadelphia, Pa, USA, 2nd edition, 2002.

[2] Cabbar F, Nur RB, Dikici B, Canpolat C, Capar GD. New bone formation by orthodontic tooth movement for implant placement. Ann Maxillofac Surg. 2016;6(2):316–8. 10.4103/2231-0746.200332. 28299281 10.4103/2231-0746.200332 PMC5343651

[3] Ribeiro GL, Jacob HB. Understanding the basis of space closure in Orthodontics for a more efficient orthodontic treatment. Dental Press J Orthod. 2016;21(2):115–25. 10.1590/2177-6709.21.2.115-125.sar. 27275623 10.1590/2177-6709.21.2.115-125.sar PMC4896290

[4] Einy S, Avezov K, Aizenbud D. Geminated maxillary incisors: the success of an orthodontic conservative approach: 15 years follow-up study. Appl Sci. 2022;12:1389. 10.3390/app12031389.

[5] Mahendra L, Govindarajan S, Jayanandan M, Shamsudeen SM, Kumar N, Madasamy R. Complete bilateral gemination of maxillary incisors with separate root canals. Case Rep Dent. 2014;2014:425343. 10.1155/2014/425343. 25254121 10.1155/2014/425343 PMC4164315

[6] Duncan WK, Helpin ML. Bilateral fusion and gemination: a literature analysis and case report. Oral Surg Oral Med Oral Pathol. 1987;64:82–7. 3475662 10.1016/0030-4220(87)90121-6

[7] Rajeswari M, Ananthalakshmi R. 2011. Gemination-case report and review. Indian Journal of Multidisciplinary Dentistry

[8] Crawford NL, North S, Davidson LE. Double permanent incisor teeth: management of three cases. Dent Update. 2006;33(10):608–10. 17209535 10.12968/denu.2006.33.10.608

[9] Shrivastava S, Tijare M, Singh S. Fusion/double teeth. J Ind Acad Oral Med Radiol. 2011;23:S468–70.

[10] Williams S, Andersen CE. The morphology of the potential class III skeletal pattern in the growing child. Am J Orthod. 1986;89(4):302–11. 3457529 10.1016/0002-9416(86)90052-7

[11] Park JU, Baik SH. Classification of Angle class III malocclusion and its treatment modalities. Int J Adult Orthodon Orthognath Surg. 2001;16(1):19–29. 11563392

[12] Marco R. Missing teeth in the smile area: space closure in all malocclusions looking for long term health, esthetics and function. Sem Orthodont. 2020;26:1.

[13] Rosa M, Zachrisson BU. Integrating space closure and esthetic dentistry in patients with missing maxillary lateral incisors. J Clin Orthod. 2007; 41(9): 563–73; 17921603

[14] Czochrowska ,E.M.,Skaare,A.B.,Stevnik A, Zachrisson, B.U. Outcome of orthodontic space closure with a missing maxillary central incisor 10.1016/s0889-5406(03)00054-4 12806336

[15] Zachrisson BU. Improving orthodontic results in cases with maxillary incisors missing. Am J Orthod. 1978;73(3):274–89. 10.1016/0002-9416(78)90134-3. 274073 10.1016/0002-9416(78)90134-3

[16] Wehrbein H, Merz BR, Diedrich P, Glatzmaier J. The use of palatal implants for orthodontic anchorage. Design and clinical application of the orthosystem. Clin Oral Implants Res. 1996 Dec;7(4):410–6 10.1034/j.1600-0501.1996.070416.x 9151610

[17] De Clerck H, Cevidanes L, Baccetti T. Dentofacial effects of bone-anchored maxillary protraction: a controlled study of consecutively treated class III patients. Am J Orthod Dentofacial Orthop. 2010;138(5):577–81. 21055597 10.1016/j.ajodo.2009.10.037 PMC3033914

[18] Rosa M. Essay I: orthodontic edentulous space closure in all malocclusions. Int J Esthet Dent. 2020;15(Suppl 1):S14–31. 32467932

